# Impact of Antipsychotic Use on Readmission Rates in Children and Adolescents With Autism Spectrum Disorder and Irritability

**DOI:** 10.7759/cureus.22361

**Published:** 2022-02-18

**Authors:** Sindhura Kompella, Angela Vittori, Jessica Kroin, Shivani Kaushal, Sara Khan, Samuel Neuhut

**Affiliations:** 1 Psychiatry, Aventura Hospital and Medical Center, Aventura, USA; 2 Dr. Kiran C. Patel College of Allopathic Medicine, Nova Southeastern University, Davie, USA

**Keywords:** 30-day readmission rate, aggression in children, aripiprazole, risperidone, hospital readmission, 90 days, 60 days, irritability, antipsychotic medication, autism spectrum disorder (asd)

## Abstract

Background

Risperidone and aripiprazole have been established as standard pharmacological treatments for irritability and associated aggressive behaviors in individuals with autism spectrum disorder (ASD), and are the only drugs approved by the United States Food and Drug Administration for those purposes. However, the rates of readmission with the use of these drugs in the pediatric population have not been studied, leaving a gap in the knowledge of antipsychotic effects. Readmission rates are a valuable metric of treatment efficacy that also reflect the financial burden, morbidity, and medical complications associated with multiple hospitalizations.

Methodology

A retrospective study was conducted in 65 Hospital Corporation of America Healthcare hospitals within the United States from 2016 to 2019. Patients aged 6-17 years with a diagnosis of ASD with irritability were included. The primary outcome was 30-, 60-, and 90-day readmission rates. Chi-square tests of independence and post-hoc analyses were used to assess the relatedness between readmission rate and antipsychotic use, as well as the type of antipsychotic medication if used. A binary regression analysis was used to analyze the relationship between demographic characteristics and readmission rate in this population. Patients on antidepressants, anxiolytics, or medications primarily used as mood stabilizers were excluded from the study to reduce confounding effects of such medications.

Results

A total of 2,375 patients aged 6-17 years were admitted for irritability and a diagnosis of ASD. In total 323 (13.8%) patients were readmitted from this group within 30 days of discharge. After controlling for age, sex, and gender, the use of antipsychotic medication was found to decrease 30- and 90-day readmission rates with an odds ratio of 1.2 to 1.4 times compared to no antipsychotic use (p < 0.04). In patients with autism not on antipsychotics, regression analysis revealed that older age (p = 0.0471) and White race (p = 0.0471) were associated with 30-day readmission (a = 0.05). For these patients, race was also significantly associated with 60-day (p = 0.0494) and 90-day (p = 0.0416) readmission rates. In patients with autism on either risperidone or aripiprazole, age (p = 0.0393) and race (p = 0.0316) were significantly associated with 30-day readmission rate.

Conclusions

Antipsychotic use reduced readmission rates within 30 days and 90 days in patients with irritability and ASD. Additionally, oral aripiprazole and oral risperidone were found to be equally effective in reducing the 30-day readmission rate, and neither was superior in comparison to the other in 30-, 60-, or 90-day readmission rates. The reduced 30- and 90-day readmission rates seen in our study with the use of antipsychotic medications emphasize the importance of antipsychotic use for individuals with ASD and irritability, even if the antipsychotic is not risperidone or aripiprazole. Groups who can particularly benefit from antipsychotic use include individuals who are refractory to first- and second-line therapies, such as behavioral interventions, or for those who present with persistent and serious risk of harm to themselves or others. Additionally, the use of antipsychotic medications in this scenario may reduce hospitalizations within 30 days of discharge, allowing reduction of the financial and emotional strain associated with these readmissions.

## Introduction

Through multiple randomized control trials and subsequent meta-analyses, risperidone and aripiprazole have been established as standard pharmacological treatments for irritability and associated aggressive behaviors in individuals with autism spectrum disorder (ASD), and these are the only drugs approved by the United States Food and Drug Administration (FDA) for those purposes. In addition, these drugs have proven effective at ameliorating attention-deficit hyperactivity disorder (ADHD) symptoms in the ASD population, further supporting their widespread use to reduce the behavioral difficulties that these patients experience [1]. However, the significant side effects associated with risperidone and aripiprazole have prompted the more widespread practice of reserving these drugs for individuals who are refractory to first- and second-line therapies, such as behavioral interventions, or for those who present with persistent and serious risk of harm to themselves or others [2]. Other medications that have yielded moderate to large effect sizes in the treatment of irritability and aggression in ASD, some of which have milder side effect profiles, include N-acetylcysteine, clonidine, methylphenidate, and tianeptine [1].

In studies comparing risperidone and aripiprazole head-to-head, the drugs appear to have similar effects in assessments of symptom reduction using behavioral checklists and psychiatric scales [3]. However, the rates of readmission in children treated with risperidone or aripiprazole have not yet been studied, demonstrating a gap in information that should be considered in the adverse effect profiles of these drugs.

Psychiatric readmission rates are a useful metric to determine the extent of severe psychiatric symptoms and efficacy of treatment, which are especially important to consider in patient populations with high rates of comorbid psychiatric disorders (such as patients with intellectual disability, anxiety disorder, mood disorder, etc.). Factors associated with psychiatric readmission have been studied in general populations of children and adolescents admitted to psychiatric inpatient units. In one meta-analysis of psychiatric hospital readmissions of children and adolescents, there was an overall 13.2% readmission rate for youths, with readmission correlated to suicidal ideation at first admission, diagnosis of a psychotic disorder, prior hospitalization, and discharge to residential treatment [4]. Other studies have also identified greater symptom severity, clinical diagnosis of affective disorders, poor family functioning, and longer lengths of index hospital stay as risk factors for psychiatric readmission in children and adolescent patients [5-7]. However, investigations of psychiatric readmissions in individuals with autism are scarce in the current literature and have been limited to one study in an adult population. Sheehan et al. identified intellectual disability, male gender, younger age, and previous admission as factors associated with a higher likelihood of psychiatric admission in adults with autism. Diagnoses of schizophrenia spectrum disorder, affective disorder, or personality disorder were also associated with higher psychiatric admission rates [8]. Generally, increased clinical severity appears to correlate with the risk of psychiatric readmission, suggesting that populations with extensive clinical needs, such as those with ASD and irritability, may be at an increased risk of readmission. Identifying such risk factors can be extremely valuable to optimize treatment for populations more vulnerable to poor outcomes, highlighting the importance of this kind of investigation in children with autism. In addition to signifying various potentially modifiable aspects of patient treatment, readmissions themselves are negative experiences for patients and their families, inflicting further emotional and financial burdens [9-11].

In this study, we assess the utility of antipsychotic medications for treating youths with ASD and irritability in the context of psychiatric readmission rates. In addition to antipsychotic medication use, we explore factors associated with increased psychiatric readmission rates in children and adolescents with ASD, for which there remains a knowledge gap. Our research compares 30-, 60-, and 90-day readmission rates in patients with ASD and irritability among different types of antipsychotic use, namely, risperidone, aripiprazole, other antipsychotics (including quetiapine, haloperidol, olanzapine, and ziprasidone), and no antipsychotic use.

## Materials and methods

This is a retrospective analysis of data from the Hospital Corporation of America (HCA) Healthcare Enterprise-level database from 2016 to 2019. Inclusion criteria included demographics such as gender (male or female), age (6-17 years), and ethnicity (White, African American, and Other). Inpatient discharge and readmission data were obtained using the keywords “irritability” and “autism spectrum disorder.” Criteria from the fifth edition of the Diagnostic and Statistical Manual of Mental Disorders and the International Classification of Diseases, Tenth Revision code for autistic disorder (F84.0) were used to determine patients with a diagnosis of ASD.

Data on the presence or absence of antipsychotic use were also queried. Four antipsychotic medication groups were delineated in this dataset: risperidone; aripiprazole; “other antipsychotic,” which included quetiapine, olanzapine, and ziprasidone; and “no antipsychotic medication.” Patients using antidepressants, mood stabilizers, anxiolytics, or haloperidol lactate were excluded from the study.

The hospitalization indices collected for readmission data were day one, day 30, day 60, and day 90 after hospitalization of patients being treated for irritability in ASD. Urbanization codes were analyzed using the Esri Demographics Tapestry Segmentation system. Urban and rural geographical localities were further divided into sub-geographic locations consisting of metro cities, principal urban centers, rural, semirural, suburban periphery, and urban periphery. Insurance status data were also analyzed and divided into private, government, or no insurance. Insurance was used as a measure of socioeconomic status.

The data on antipsychotic use and readmission were analyzed using chi-square tests of independence to assess the relatedness between 30-, 60-, and 90- readmission rates and the use of antipsychotic versus no medication in patients with ASD and irritability. For results showing an association between antipsychotics used and readmission rates, post-hoc chi-square analysis was utilized to determine which antipsychotic types were associated with readmission at a 95% confidence level. A binary regression analysis was used to analyze the relationship between demographic characteristics and readmission rates in this population group.

## Results

Our study identified a total of 2,375 patients aged 6-17 years with irritability and ASD in the HCA database from 2016 to 2019 who met the inclusion criteria. In patients with autism with no antipsychotic medication, age (p = 0.0471) and race (p = 0.0098) were found to have an association with 30-day readmission at a 95% confidence level (α = 0.05). Specifically, patients with a one-year increase in age were more likely to be readmitted in 30 days, with an estimated regression coefficient of 0.0328 (i.e., patients were more likely to be readmitted if their age was higher). Meanwhile, White patients were more likely to be readmitted in 30 days than patients of race labeled “Other” (i.e., not White or African American), with an estimated regression coefficient of 0.2234. The variable of race (White) alone was found to have an association with 60- and 90-day readmissions (p = 0.0494, p = 0.0416, respectively) for patients not taking any antipsychotic medication at a 95% confidence level (α = 0.05), with an estimated regression coefficient of 0.1469 for 60 days and 0.1434 for 90 days. Thus, White patients with autism aged 6-17 years are more likely to be readmitted within 60 and 90 days of hospitalization than patients of race labeled “Other” when not taking antipsychotic medications.

The associations of age and race with readmission rates differed in patients with ASD who were on either risperidone or aripiprazole. In these patients, the following variables were found to have an association with only 30-day readmission at a 95% confidence level (α = 0.05): race (White) (p = 0.0316), with an estimated regression coefficient of 0.1916, and age (p = 0.0393), with an estimated regression coefficient of 0.0353 for each one-year increase. Hence, White patients with autism on either risperidone or aripiprazole were more likely to be readmitted in 30 days than those of a race other than White or Black. Individuals with autism on either risperidone or aripiprazole were also more likely to be readmitted in 30 days with each one-year increase in age. One may be 95% confident that a patient’s odds are between 1.002 and 1.071 times as likely to be readmitted in 30 days compared to when they were one year younger. However, no significant associations were found for age or race with 60- and 90-day readmission rates in patients with autism who were taking either risperidone or aripiprazole.

As shown in Table 1 and Table 2, aripiprazole, risperidone, and other medication groups are equally comparable in reducing readmission rates within 30 days of discharge (p < 0.136). Notably, the patients on either risperidone or aripiprazole and other medication groups were both found to be significant in reducing readmission rate within 30 days in comparison to the no medication group (p < 0.007 and p < 0.01, respectively).

**Table 1:** Chi-square analysis for 30-day readmission rates in patients with irritability and autism spectrum disorder. PO = oral formulation; 30-day readmission: 0 = no readmissions versus 1 = readmissions; % readmitted = percentage of antipsychotic medication group readmitted to the psychiatric inpatient unit; Chi square P-value for 30-day readmission rates = P-value for chi-square test of association between 30-day readmission rate and antipsychotic medication groups.

Antipsychotic name (PO)	30-day readmission		Total	% readmitted
	0	1		
Aripiprazole (Ari)	231	38	269	14.13
Risperidone (Ris)	242	33	275	12.00
Other medication	203	47	250	18.80
No medication	1,247	185	1,432	12.91
Chi-square P-value for 30-day readmission rates: 0.0125				

**Table 2:** P-values for pairwise comparison in post-hoc chi-square analysis of 30-day readmission rates. Ris/Ari = patients using either risperidone or aripiprazole P-value of 0.0072 indicates a significant reduction of 30-day readmission rates in the Ris/Ari group compared to the no medication group. P-value of 0.0167 indicates a significant reduction of 30-day readmission rates in the other medication group compared to the no medication group.

Pair compared	P-value
No medication vs. Ris/Ari	0.0072
Other vs. Ris/Ari	0.1364
No medication vs. other	0.0167

Table 3 summarizes the results for 60-day readmission rates in patients with irritability and ASD. Overall, chi-square analysis shows no significant differences in 60-day readmission rates among the risperidone, aripiprazole, no medication, and other medication groups at the 95% (α = 0.05) confidence limit (p < 0.0661).

**Table 3:** Chi-square analysis for 60-day readmission rates in patients with irritability and autism spectrum disorder. PO = oral formulation; 60-day readmission: 0 = no readmissions versus 1 = readmissions; % readmitted = percentage of antipsychotic medication group readmitted to the psychiatric inpatient unit; Chi-square P-value for 60-day readmission rates = P-value for chi-square test of association between 60-day readmission rates and antipsychotic medication groups.

Antipsychotic name (PO)	60-day readmission		Total	% readmitted
	0	1		
Aripiprazole (Ari)	221	48	269	17.84
Risperidone (Ris)	227	48	275	17.45
Other medication	193	57	250	22.80
No medication	1,171	261	1,432	18.23
Chi-square P-value for 60-day readmission rates = 0.0661				

Table 4 summarizes the results for 90-day readmission rates in patients with irritability and ASD. Overall, chi-square analysis shows a significant reduction in 90-day readmission rates for the risperidone/aripiprazole groups in comparison to the no medication group (p < 0.0057), while the other medication groups did not have significantly different readmission rates compared to the no medication groups (p < 0.0667) (Table 5). There are also no significant differences in readmission rates found among risperidone, aripiprazole, and other medication groups (p < 0.80).

**Table 4:** Chi-square analysis for 90-day readmission rates in patients with irritability and autism spectrum disorder. PO = oral formulation; 90-day readmission: 0 = no readmissions versus 1 = readmissions; % readmitted = percentage of antipsychotic medication group readmitted to the psychiatric inpatient unit; Chi-square P-value for 90-day readmission rates = P-value for chi-square test of association between 90-day readmission rate and antipsychotic medication groups.

Antipsychotic name (PO)	90-day readmission		Total	% readmitted
	0	1		
Aripiprazole (Ari)	210	59	269	21.93
Risperidone (Ris)	214	61	275	22.18
Other medication	185	65	250	26.00
No medication	1,135	297	1,432	20.74
Chi-square P-value for 90-day readmission rates = 0.0137				

**Table 5:** P-values for pairwise comparison in post-hoc chi-square analysis of 90-day readmission rates. Ris/Ari = patients using either risperidone or aripiprazole P-value of 0.0057 indicates a significant reduction of 90-day readmission rate in the Ris/Ari group compared to the no medication group.

Pair compared	P-value
No medication vs. Ris/Ari	0.0057
Other vs. Ris/Ari	0.8600
No medication vs. other	0.0667

Binary regression analysis was performed to analyze the association of demographics and medication use with 30-, 60-, and 90-day readmission at a 95% confidence level (α = 0.05). Factors found to be significantly correlated with readmission rates included White race (p = 0.0316), age (p = 0.0393), and antipsychotic versus no antipsychotic use (p = 0.041).

## Discussion

Age

In patients aged 6-17 years, as age increased by one year, 30-day readmission rates were found to be significantly higher whether or not the groups were on an antipsychotic, i.e., risperidone, aripiprazole, or no antipsychotic medication. Age was not found to be a significant variable for 60- and 90-day readmission rates in either the antipsychotic medication group or the no medication group. This may be due to the presentation of aggressive characteristics, which may increase with age due to disease severity. One particular manifestation of aggression, self-injurious behavior, is more likely to persist over time (i.e., at an older age) in children with ASD who are non-verbal, have lower ability, and have higher levels of overactivity, impulsivity, and repetitive behavior (i.e., more severe symptoms of ASD) [12]. The severity of the illness is usually controlled if appropriate psychopharmacologic intervention and behavioral therapy are provided in a timely manner; however, even with the appropriate management, some illnesses are more complex due to comorbid illnesses such as ADHD, which is highly prevalent in this population group as patients grow older [13]. Additionally, mood disorders and intellectual disability may lead to increased rehospitalization rates in this population group as complications of these disorders have been shown to increase with age [14].

Moreover, age plays a role in behavioral therapy for socialization deficits and behavioral patterns in this population group. Such therapeutic interventions usually require more time for older patients because younger patients are more likely to be able to learn new coping strategies compared to older patients, who may have already formed maladaptive behaviors [15,16]. Additionally, as patients with ASD grow older, medications may take longer to work in this population group due to increased tolerance from more likely use of multiple prior antipsychotic medications for irritability, which may result in more readmissions due to reduced effects on symptom control within 30 days of discharge [17]. Thus, it is important to be aware of these risk factors because as this population group increases in age, they are more likely to be readmitted within 30 days of discharge, increasing length of stay at the hospital and contributing to a greater financial and emotional burden.

Race

According to our study, White individuals are more likely to be readmitted within 30 days of discharge. Of the total 2,336 patients with ASD and irritability in this study, 1,300 (55.6%) were White, 548 (23.4%) were Black, and 410 (17.5%) were other races. Because 55.6% of the sample was White, the probability that White children and adolescents are followed up and readmitted is magnified. Our sample is also consistent with how the demographics of ASD are described in the literature; ASD is more prevalent in White children in comparison with other races. Different studies have tried to explain this demographic distribution. White patients, especially those with severe ASD and intellectual disability, tend to be diagnosed earlier than children from other races. Furthermore, cultural barriers and racial differences in parent reports to healthcare providers can delay ASD diagnoses in non-White children [18].

White patients in this study’s patient population are more likely to be readmitted in 30 days, but there is no difference in readmission at 60 and 90 days after discharge. We present various potential theories for this finding. Our study is limited to the access of medication reconciliation, start date, and specifications of first admissions during the established period of time of this study. Therefore, if this was a first admission and medications were started for the first time, the diagnosis, adherence to medication, and management of side effects could have been challenging for some patients and families in the first few weeks. Consequently, these could increase the probability of non-compliance and relapse of symptoms in the first 30 days of discharge. Moreover, according to Coury et al., non-White and Latino children use psychotropic medications at lower rates than White children [19]. Caregivers of Black children report denial, shame, and stigma regarding ASD in the Black community and, relatedly, family members of individuals with intellectual disability are especially susceptible to negative responses and treatment from their extended family and community in non-Western cultures [20,21]. The potential loss of social support with the label of a mental illness in non-Western and non-White communities can prevent parents from seeking out a diagnosis, interfering with the identification and treatment of these children who may require assistance for developmental delay or related issues. Insurance and language barriers in minority groups can also play an important role in the health access and reassessment of children with ASD. Services for ASD are underutilized by racial and ethnic minorities, with potential contributing factors including disparities in healthcare access, mental illness stigma among minority groups, and a lack of culturally informed ASD education for parents [22-24]. This underrepresentation of racial minorities in treatment contexts may also contribute to the racial differences in our study’s treatment-focused results, as well as to broader patterns in ASD racial demographics.

Aripiprazole versus risperidone for ASD

Previous studies have shown that psychopharmacology is effective in the treatment of autism. Evidence for antidepressants, mood stabilizers, and anxiolytics shows weak efficacy; however, there is strong evidence for symptom improvement with psychostimulants, noradrenergic reuptake inhibitors, alpha-adrenergic agonists, and antipsychotics. Among antipsychotics, the atypical antipsychotics risperidone and aripiprazole are currently the only FDA-approved medications for the treatment of irritability and aggressive behaviors in individuals with ASD. While the efficacy of such medications has been proven, we hoped to investigate and compare their outcome success in the context of readmission rates due to the many negative implications of hospital readmissions in patients with ASD [6,8]. Approximately 20% of ASD patients exhibit irritability or aggressive behaviors, which can result in an increase in hospitalization rates [1].

This study compared aripiprazole and risperidone intervention groups for patients with irritability in ASD to learn whether either of these medications is superior in reducing readmission rates in these populations. However, our results showed that patients in groups that received either risperidone or aripiprazole were equally efficacious in reducing readmission rate within 30 and 90 days of discharge, supporting previous research showing similarity between the two medications on other measures of efficacy [25]. Moreover, in comparison to no medications, the use of antipsychotic medications reduced 30- and 90-day readmission rates. This is especially significant because irritability and aggressive behaviors are the most common reasons for hospitalizations in patients with ASD within 30 days of discharge. Our research shows that the use of risperidone or aripiprazole can have an impact on 30- and 90-day readmission rates in patients with ASD and irritability, decreasing the length of stay, rehospitalizations, morbidity, and financial burden [2]. The lack of significant effect on readmission rates at 60 days may reflect attenuation of time-related factors contributing to rehospitalization. For example, rehospitalization closer to hospital discharge may reveal certain difficulties with the transition that are exacerbated by the lack of an antipsychotic regimen to manage irritability, while readmission at 90 days may be a function of difficulties compounded over a longer time period of dysregulated behavior not managed by standard treatment with aripiprazole or risperidone.

Other antipsychotics versus no antipsychotics for ASD

At the 30-day readmission time point, the group with no medications had significantly greater readmission rates than the group receiving other antipsychotics besides aripiprazole or risperidone, while there was no significant difference in readmission rates between these groups at the 90-day time point. A possible explanation for this trend could be related to the fact that risperidone and aripiprazole have the strongest evidence for reducing irritability symptoms in children with ASD, and are often first-line for these patients due to their long-term efficacy [1]. The alternative antipsychotic medications used in some of the patients in this sample, such as haloperidol, quetiapine, and ziprasidone, have been shown to be less efficacious and have more adverse side effect profiles and diminished treatment responses than risperidone or aripiprazole in children with ASD and irritability [25,26]. However, in a shorter time period (i.e., within 30 days of hospital discharge), the effects of alternative antipsychotics on 5-hydroxytryptamine and dopamine, and decreased amount of time for negative side effects to develop, likely contributed to the alleviation of behavioral symptoms, accounting for the difference in readmission rates when no antipsychotic was used [26-28]. Thus, the use of one of the less efficacious antipsychotics in the “other antipsychotic” group in this study may have contributed to the eventual decline in symptomatic benefit and convergence with readmission outcomes for patients with no antipsychotic treatment. Furthermore, while fewer patients on antipsychotics typically relapse compared to those on placebo, they also often experience more adverse effects than placebo groups [29]. Finally, non-adherence in refilling prescriptions or difficulty in finding long-term insurance coverage for medication after discharge may contribute to similar outcomes between no antipsychotic use and alternative antipsychotic use. The balance of various factors such as these could potentially contribute to the variable readmission outcomes over time for children on antipsychotics besides risperidone and aripiprazole, as well as their overlap with those of patients not taking antipsychotics.

Limitations

Limitations of our study include a small sample size for medication intervention groups when divided by each antipsychotic in comparison to the group with no medication. Another limitation is that this dataset was obtained solely from HCA hospitals; even though this sample was not geographically limited, the use of data from a singular organization warrants further analysis in other facilities in the future. To better understand contributing factors to rehospitalization in children with ASD, forthcoming studies can also investigate concurrent use of other psychotropic medications combined with antipsychotics used to treat comorbid conditions in this population. Furthermore, randomized placebo trials with outcome measures of readmission rates could more directly measure the efficacy of specific psychotropic medications on functioning in ASD. Comparing readmission rate information with standardized measures of specific ASD symptomatology would provide even more insights into the direct effects of these medications on children. Such focused investigations can significantly improve the outcomes and wellness of patients with ASD and their families by helping to identify risk factors and decrease the financial, emotional, and health burden of hospital readmissions.

## Conclusions

The variables of the White race and increased age were found to be significant predictors of readmission within 30, 60, and 90 days of hospital discharge. Additionally, both risperidone and aripiprazole were equally efficacious in reducing 30- and 90-day readmission rates in patients with irritability and ASD. Importantly, the use of antipsychotics (i.e., risperidone, aripiprazole, and an “other” group containing quetiapine, haloperidol, olanzapine, and ziprasidone) reduced 30-day readmission rates in comparison to no antipsychotic medication use. Therefore, our study shows the importance of the use of antipsychotics for individuals who are refractory to first- and second-line therapies (such as behavioral interventions, speech therapy, and occupational therapy), or for those who present with persistent and serious risk of harm to themselves or others via aggressive behaviors and irritability.
